# Circumferential periosteal block versus hematoma block for the reduction of distal radius and ulna fractures: a randomized controlled trial

**DOI:** 10.1007/s00068-022-02078-8

**Published:** 2022-08-18

**Authors:** Ali Lari, Ali Jarragh, Mohammad Alherz, Abdullah Nouri, Mousa Behbehani, Naser Alnusif

**Affiliations:** 1 AlRazi Orthopedic Hospital, AlSabah Medical Region, Kuwait City, Kuwait; 2 Department of Orthopedic Surgery, Jaber Alahmed Alsabah Hospital, Kuwait City, Kuwait; 3 Department of Anatomy, Trinity College Dublin, Dublin, Ireland

**Keywords:** Distal radius fracture, Hematoma block, Periosteal block, Analgesia, Reduction, Nonoperative management

## Abstract

**Purpose:**

To assess the analgesic efficacy of the circumferential periosteal block (CPB) and compare it with the conventional fracture hematoma block (HB).

**Methods:**

This study was a prospective single-center randomized controlled trial performed in a national orthopedic hospital. Fifty patients with displaced distal radius (with or without concomitant ulna) fractures requiring reduction were randomized to receive either CPB or HB prior to the reduction. Pain was sequentially measured using the visual analogue scale (VAS) across three stages; before administration of local anesthesia (baseline), during administration (injection) and during manipulation and immobilization (manipulation). Further, the effect of demographic factors on the severity of pain was analyzed in multivariate regression. Finally, complications and end outcomes were compared across both techniques.

**Results:**

Patients receiving CPB experienced significantly less pain scores during manipulation (VAS = 0.64) compared with HB (VAS = 2.44) (*p* =  < 0.0001). There were no significant differences between groups at baseline (*P* = 0.55) and injection (*P* = 0.40) stages.

**Conclusion:**

The CPB provides a superior analgesic effect over the conventional HB with no documented complications in either technique.

**Level of evidence:**

Therapeutic Level II.

**Supplementary Information:**

The online version contains supplementary material available at 10.1007/s00068-022-02078-8.

## Introduction

Fractures of the distal radius and ulna are among the most common in orthopedic trauma [1]. They are linked with a bimodal distribution of high and low-energy trauma in the younger and older population, respectively [1]. Geographic and provider-dependent variability in operative versus non-operative trends exist, reflecting a lack of a clear consensus or superiority of particular treatment modalities [2, 3].

Initial management often entails closed reduction and manipulation followed by immobilization, which if successful, may deter further operative management. Adequate analgesia is essential during reduction, as painful manipulation may hinder patient cooperation and preclude successful reduction [4]. Thus, various anesthetic techniques have been implemented. These notably include hematoma blocks (HB), intravenous regional anesthesia (Bier’s block), brachial plexus blocks and procedural sedation (opioids and sedatives). Hematoma blocks are widely utilized due to their simplicity. However, they require the presence of a hematoma and data has demonstrated variable analgesic efficacy [5–7]. The potential for systemic complications and the resultant need for hospital admission and monitoring limits the use of procedural sedation and Bier’s blocks in accordance with capacity and resources [7].

An ideal analgesic technique should result in safe, easy, and painless fracture manipulation. In 2015, Tageldin et al. described a novel circumferential periosteal block (CPB) technique for the reduction of radius and ulna fractures [8]. The study reported painless reductions with no documented complications. In this randomized controlled trial, we assess the efficacy of the CPB technique compared to the conventional hematoma block, with the null hypothesis being that the two block techniques are equally effective for pain relief in the reduction of the distal radius and ulna fractures.

## Patients and methods

### Study design

This was a single center, prospective randomized controlled trial. Informed consent was obtained from eligible patients prior to their inclusion in the study. Plain radiographs were reviewed and the Frykman classification was utilized to classify their fractures. The study was conducted in accordance with the 1964 Helsinki Declaration, approved by our local ethical committee board (UID 1733/2021) and registered on the Research Registry (UIN: 6639). This study adheres to the appropriate CONSORT guidelines.

### Study population

Eligible patients were over 12 years of age presenting acutely for the first time with a displaced distal radius fracture (in the presence or absence of a concomitant distal ulnar fracture) requiring reduction via manipulation. Exclusion criteria entailed known allergy to local anesthesia, multiple fractures, polytrauma, head trauma, unconsciousness, open fractures, neurovascular deficit, any evidence of compartment syndrome or ipsilateral upper limb fractures precluding effective reduction or analgesia. All included participants had presented to the emergency department within 6 h of injury, to ensure the presence of a viable hematoma. The sample size calculation for this study was based on the repeated-measures ANOVA design for two groups (HB vs CPB) measured at three observations (baseline, injection, and manipulation). A study with an effect size of 0.5 and a power of 95% required a total sample of 38 to test the association at a 5% alpha level. The power calculation was carried out using G*power 3.1.9. A total of 50 patients were included in the study.

### Randomization

Patients were selected consecutively in the emergency department of a tertiary orthopedic center if they met the inclusion criteria. Patients were block randomized to receive either HB or CPB by computer-generated lists equally distributed to two trained orthopedic surgeons.

### Techniques [8]

Patients were positioned supine with the elbow extended on a dressing table. Precautionary measures including oxygen, intravenous access and intra-lipid emulsion were available. The affected arm was draped and sterilized. For both techniques, lidocaine 1% without epinephrine was used.

For the CPB, a 10-ml syringe with a (25G) needle was used to infiltrate the subcutaneous tissue under an aseptic technique on the radial aspect of the radius approximately 6 cm proximal to the wrist joint (2–3 cm from the fracture). Once the entire radial aspect (subcutaneous and periosteal) is infiltrated, the needle is changed to a (22G) needle. The next injection followed the parallel plane of the dorsal and volar surfaces of the radius, ensuring contact with bone whilst advancing to avoid any soft tissue or neurovascular structures. Rolling of the skin allowed for a single injection to access both surfaces. In the presence of a concomitant ulnar fracture, the process is repeated on the ulnar aspect. Finally, manipulation was performed 15 min after the block, whilst recording the required data.

The hematoma block was performed in the traditional way. A 22G needle is inserted on the dorsal aspect of the radial fracture, aspiration of the hematoma to confirm location, followed by subsequent injection of lidocaine within the hematoma. Techniques to minimize pain on injection of local anesthesia were utilized in both blocks [9].

### Outcome assessments

The primary outcome was pain measured using the visual analogue scale (VAS 0–10). Measurements were taken across three stages; before administration of local anesthesia (baseline), during administration (injection) and during manipulation and immobilization (manipulation). Post-reduction radiographs were obtained. Patients were subsequently monitored in the emergency department for 1 h to assess for any acute complications. Further, patients were evaluated for secondary outcomes according to age, sex, definitive treatment (within 6 weeks), Frykman classification, radiographic assessment, need for re-manipulation and the presence of any intervention-related complication. Complications were defined as any procedure-related adverse effects including; distal sensory or motor deficit, vascular deficit, local infection, nausea, vomiting, respiratory distress and weakness derived from tendon injuries.

### Statistical analysis

**Fig. 1:**
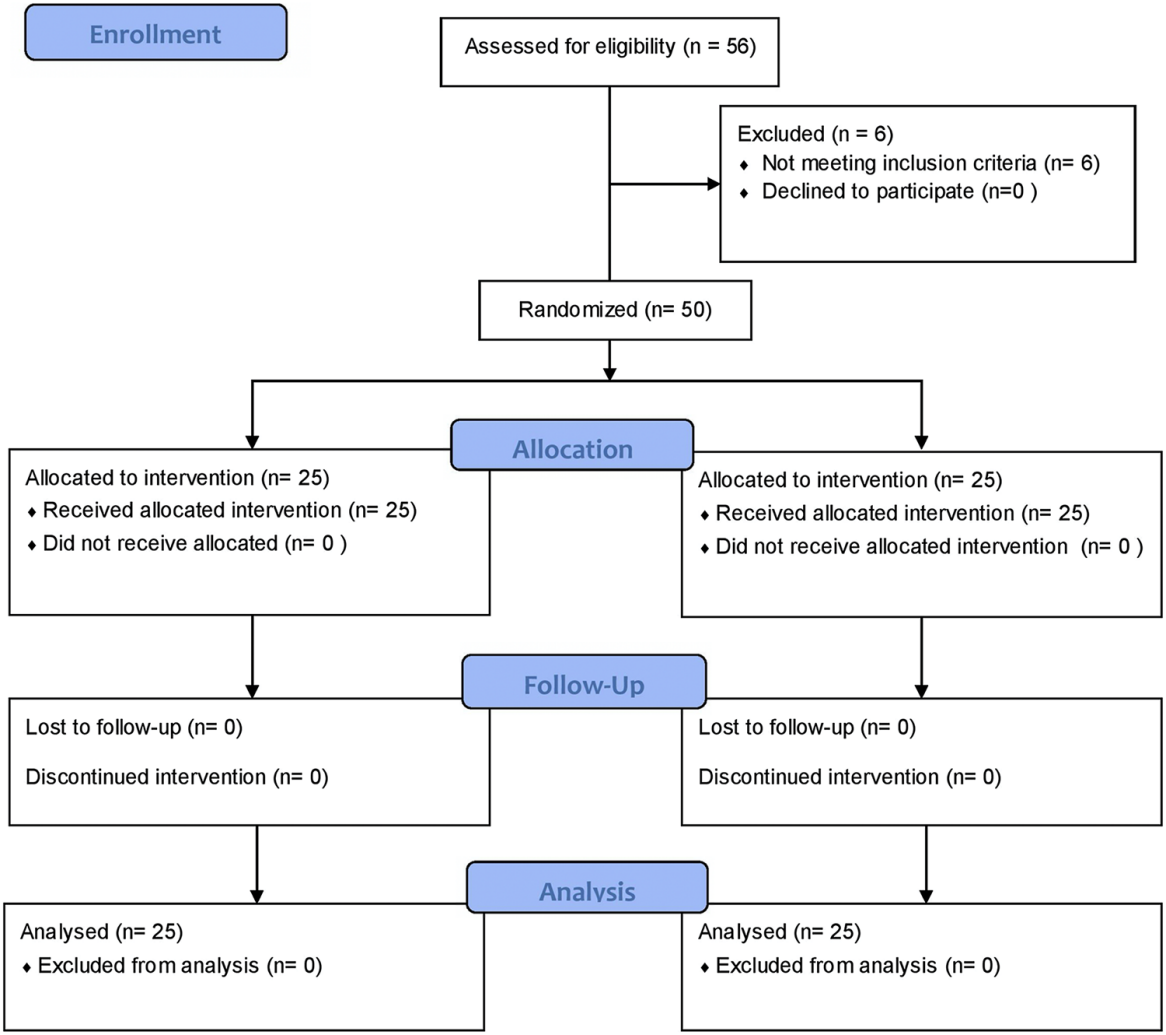
CONSORT Flowchart displaying enrolment, allocation, follow up and analysis of participants

Data analysis was performed using Minitab 19 (Minitab LLC, PA USA) and Graphpad Prism 9. Descriptive statistics shown in Table 1 for the 25 participants in each group employ the median, interquartile range, frequency and percentage of patients as appropriate. Differences between HB and CPB groups on demographics, fracture type/classification and outcome variables were tested using chi-squared tests (*χ*^2^) for categorical variables and the Mann–Whitney test for age due to its non-parametric distribution (Table 1). A repeated-measures two-way ANOVA was used to test for differences between the two techniques, between the three stages (baseline, injection, manipulation), as well as the interaction of the two main effects (Stage × Method) (Table 2). Post-hoc analysis for differences between the two groups at each stage employed Bonferroni’s multiple comparisons test (Fig. 1).

**Table 1:** Distribution and between-group comparisons of participants according to patient and fracture variables

Variable	Hematoma Block *n* (%)	Circumferential Block *n* (%)	χ^2^; *P* value/MWU; *P* value
Age			
Median (IQR)	41 (30.5–51.5)	44 (29.5–53)	MWU: 307, *P* = 0.9195
Sex			
Male	15 (60%)	18 (72%)	χ^2^: 3.539, *P* > 0.5505
Female	10 (40%)	7 (28%)	
Fracture			
BB	3 (12%)	5 (20%)	χ^2^: 2.624, *P* > 0.6997
DER	22 (88%)	20 (80%)	
Frykman			
1	13 (52%)	11 (44%)	χ^2^: 21.84, *P* = 0.4208
2	1 (4%)	0 (0%)	
3	9(36%)	8 (32%)	
4	1 (4%)	5 (20%)	
8	1 (4%)	1 (4%)	
Radiograph after reduction			
Accepted	23 (92%)	21 (84%)	χ^2^: 1.264, *P* > 0.6634
Equivocal	2 (8%)	4 (16%)	
Definitive treatment			
Conservative	22 (88%)	19 (76%)	χ^2^: 1.518, *P* > 0.4616
Operative	3 (12%)	6 (24%)	
Need for re-manipulation			
No	24 (96%)	24 (96%)	χ^2^: 1.208, *P* > 0.9999
Yes	1 (4%)	1 (4%)	
Complications			
No	25 (100%)	25 (100%)	χ^2^: 0.000, *P* > 0.9999
Yes	0 (0%)	0 (0%)

**Table 2:** Results of repeated-measures ANOVA measuring pain scores across stages of anesthesia

Repeated measures 2-way ANOVA	*F* (DFn, DFd)	*P* value
Stage × Method	*F* (2, 96) = 15.42	< 0.0001
Stage	*F* (1.881, 90.28) = 357.9	< 0.0001
Method	*F* (1, 48) = 5.875	= 0.0192
Subject	*F* (48, 96) = 2.268	= 0.0003

In addition, a multivariate linear regression analysis was carried out to assess the relationship of pain during manipulation as the dependent variable, with the independent variables of age, gender, operator, baseline level of pain, fracture type and Frykman classification. The regression model (Table 3) yielded an *R*^2^ of 52.3% and a *P* value of 0.0005 for the overall regression. Individual *P* values for each of the predictors along with the coefficient and 95% Confidence Intervals (95% CI) are reported in Table 3. Diagnostics on the assumptions of, linearity, homoscedasticity, and independence, were assessed using plots of the residual errors. The Anderson–Darling (*P* = 0.9520) Kolmogorov–Smirnov (*P* =  > 0.1000) and Shapiro–Wilk (*P* = 0.9864) tests confirmed normality, and the assumption of lack of multicollinearity was verified using the Variance Inflation Factor (VIF). A *P* value of less than 0.05 was considered to be statistically significant.

**Table 3:** Multiple linear regression for predictors of pain during manipulation

Variable	*P* value	Estimate	Standard error	95% CI	
				Lower	Upper
Age [years]	0.0294*	0.029	0.013	0.003	0.054
Gender [F/M]	0.7178	− 0.137	0.378	− 0.901	0.626
Fracture [BB/DER]	0.3249	1.309	1.313	− 1.347	3.966
Frykman classification [vs. 1]					
2	0.9276	− 0.166	1.817	− 3.841	3.509
3	0.1621	− 0.548	0.384	− 1.325	0.230
4	0.1193	− 2.023	1.270	− 4.592	0.546
8	0.3251	− 1.645	1.651	− 4.984	1.694
Baseline pain score	0.0349*	0.348	0.159	0.026	0.670
Operator [A/B]	0.3536	0.348	0.371	− 0.402	1.097
Block method [HB/CPB]	< 0.0001***	− 1.789	0.352	− 2.502	− 1.077

Bold indicates statistically significant values

*BB* both bone, *DER* distal end radius

## Results

Between January 2021 and June 2021, a total of 56 patients were consecutively considered for inclusion to receive either an HB or CPB (Fig. 1). Six of these patients were excluded for not meeting the inclusion criteria, of which 4 were excluded for ipsilateral upper limb fractures and 2 were excluded due to the presence of significant head trauma. Finally, the sample size of 50 patients who met the inclusion criteria was reached with 25 patients in each group.

The descriptive characteristics of our study population are displayed in (Table 1). Out of the 50 patients, the majority were males (66%) who sustained isolated distal end radius (DER) fractures (84%). The age range was 13–67 years old. Frykman classification 1 and 3 were the most common patterns encountered. The vast majority of the fractures underwent non-operative treatment after successful reduction (88%) with no significant difference between block method and outcome. Nine patients (18%) underwent operative management; 5 with percutaneous pinning and 4 with ORIF. There were no documented procedure-related complications in both utilized techniques. Two patients; one in each group, subsequently required re-manipulation. No statistical significances were observed between the distributions and end outcomes of both groups, thereby ensuring comparability.

Repeated measures 2-way ANOVA (Table 2) revealed significant main effects for both method and stage (*P*: 0.0192, < 0.0001, respectively), the latter being an indicator of achieved analgesia regardless of method. In addition, the significant interaction effect (*P* < 0.0001) indicates that the differences between block methods vary across the stages of analgesia. Post-hoc comparisons using a Bonferroni correction confirmed that a difference between block methods is only found during the manipulation stage (*P* < 0.0001). Finally, within-subject variation was also significant indicating effective control of within-subject variation (*P* = 0.0003).

**Fig. 2:**
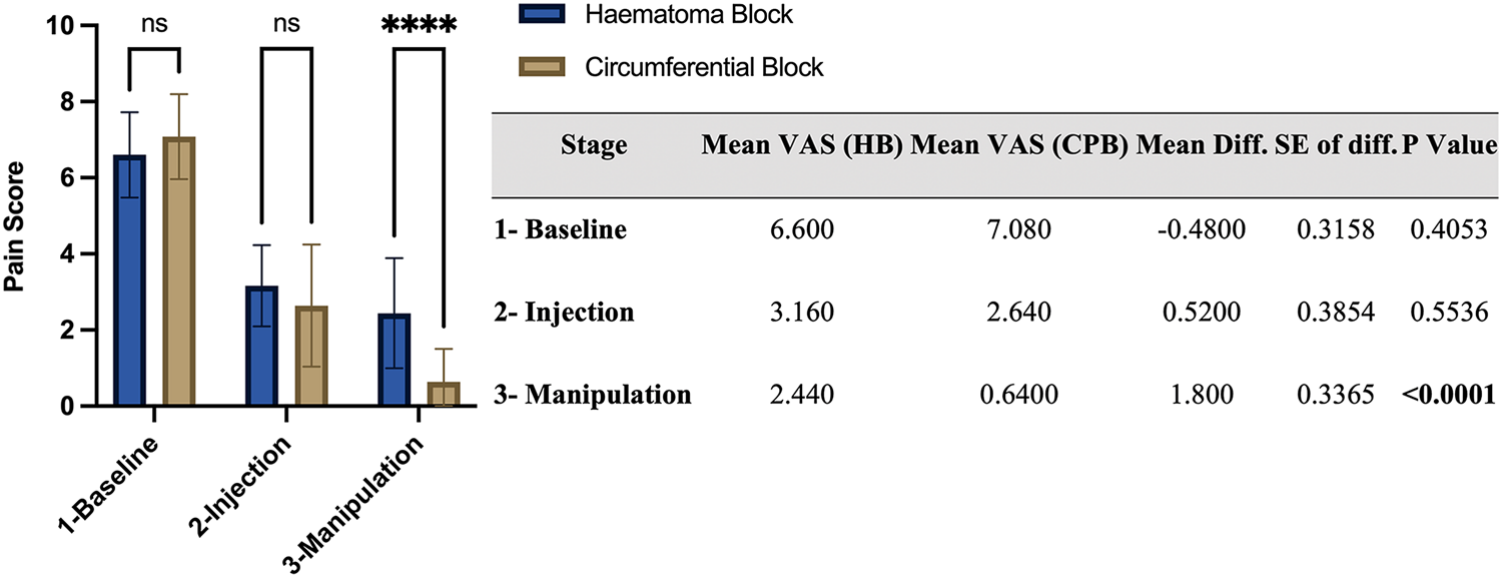
Bonferroni’s multiple comparison test for differences in pain scores between the block methods

Post-hoc analysis revealed that the only significant difference between the two groups was found to be during the manipulation stage (*P* =  < 0.0001). The lack of a difference at baseline confirms the comparability of both groups. There was no difference during the injection stage (*P* = 0.55). The mean pain score during manipulation for HB was 2.44 and for CPB 0.64 (Fig. 2). Hematoma blocks had a wider VAS range of 0–5 (SD = 1.44), whereas CPB had a narrower VAS range of 0–3 (SD = 0.86).

Predictors of pain during manipulation using multivariate regression analysis are displayed in Table 3. None of the gender, fracture types and Frykman classification were found to be significant factors in affecting the severity of pain during reduction. Notably, there was no significant operator variability in pain among block methods. Significant predictors of pain during manipulation include; increasing age (*P* = 0.029), baseline pain score (*P* = 0.0349) and block method (*P* < 0.0001). Overall, CPB reduced pain during manipulation by 1.789 points (VAS score), whilst keeping all other variables constant.

## Discussion

The management of distal radius and ulna fractures remains largely non-operative, emphasizing the necessity of adequate reduction [10]. Fracture reduction can be unpleasant for both patient and provider if pain is not sufficiently managed. This study has shown a significantly superior analgesic effect using the CPB compared to the HB, in which all reductions were described as either painless (VAS = 0) or minimally painful (VAS = 1–3). Further, no operator variability existed in both the CPB and HB.

The variability in the analgesic effect of the HB may necessitate supplementary analgesia. In our study, the HB displayed a wider, less predictable range of analgesic effect (VAS 0–5) than the CPB (VAS 0–3). The lack of predictability encountered in our study is in keeping with previous literature [5, 6, 11]. Similar to our data, Myderrizi et al. noted a mean VAS score of (2.25) [5]. Fathi et al. also described wide-ranging pain scores during the reduction of distal radius fractures after an ultrasound-guided hematoma block (numerical rating scale = 1–7) [12]. This is further reinforced in an article by Orbach et al., with a mean VAS range of (3–5.5) [6].

Yet it is worth mentioning that the degree of force in manipulation techniques is normally tailored to the nature of the fracture itself and the subsequently predicted difficulty in reduction. The latter may influence variation among pain scores in different studies. Our institutional practice of closed reduction attempts in all distal radius fractures enabled the inclusion of higher Frykman grades in our study. However, growing evidence supports a more selective approach in assessing which fractures may benefit from closed reduction [13, 14]. Fractures that possess some indication for operative management may be unlikely to benefit from closed attempts to restore acceptable radiographic parameters [15].

Further, our analysis has shown that a higher baseline pain score predicts greater pain during manipulation. This may reflect a higher sensitivity to pain in a specific subset of patients. Increasing age also exerted a statistically significant, yet inconsiderable effect on pain during manipulation.

Our results have reproduced Tageldin et al.’s [8] findings. The consistent pain scores also suggest that the technique is reproducible and requires only an adequate understanding of the technique, anatomy and administration of local anesthesia. The technique may be of particular use in cases where; hematomas are not present or difficult to access and in re-manipulations of older fractures in either the emergency or outpatient setting. In comparison to Bier’s block and procedural sedation, both techniques require fewer resources to perform, and are less likely to result in systemic complications [11, 16]. A similarly promising alternative is the wide-awake local anesthesia no tourniquet (WALANT) technique [17]. Although a potentially more experience-demanding technique compared to the CPB, its application in the painless surgical management of hand surgery and distal radius fracture fixation appears to be effective [18, 19]. Which perhaps further advocates for the value of periosteal infiltration in reducing fracture pain.

An initial concern was the risk of neurovascular injury while entering the volar surface of the radius. However, no complications of this nature arose during close follow-up and examination. The CPB also offers the hypothetical advantage of providing distance from the fracture hematoma. Unlike the hematoma block which has the theoretical risk of converting a closed fracture into an open fracture by directly accessing the hematoma, thereby increasing the risk of infection, the CPB is performed 2–3 cm proximal to the hematoma site. Future direction should focus on operator experience-dependent reproducibility of the technique. Further, larger sample sizes should aim to uncover potential complications.

This study included a limited number of operators and did not take into account experience with the technique. There was no difference in definitive outcomes after reduction, which perhaps indicates the effectiveness of analgesia may not affect end-outcomes. However, this may be an artefact of relatively small sample size, and future research may benefit from matching fracture characteristics to assess outcomes accurately. While participants were unaware of the allocated blocks during administration, there was no feasible method of blinding the operators from the interventions, which is a potential source of bias.

## Conclusion

The circumferential periosteal block offers a superior analgesic effect and a potentially equally safe alternative to the traditional hematoma block during manipulation of distal radius and ulna fractures.

## Supplementary Information

Below is the link to the electronic supplementary material.

Supplementary file1 (DOC 218 KB)

Supplementary file2 (DOCX 195 KB)
