# Supplementary material for: Circumferential periosteal block versus hematoma block for the reduction of distal radius and ulna fractures: a randomized controlled trial
Source: Eur J Trauma Emerg Surg. 2022 Aug 18;49(1):107–13. doi: 10.1007/s00068-022-02078-8 (PMC9925527; doi:10.1007/s00068-022-02078-8)
Supplement: Supplementary file 2 — Supplementary file2 (DOCX 195 KB) [file 68_2022_2078_MOESM2_ESM.docx]

**Ordinal analysis of VAS**

Mixed ordinal logistic regression (using logit link) was used to test for differences between the two techniques, between the three stages (baseline, injection, manipulation), as well as the interaction of the two main effects (Stage x Method) (Table 2). Mixed ordinal regression was used to take into account the ordinal nature of the VAS score and the correlation of VAS values obtained from each patient. Stage and method were included as main effects and patients were included as a random effect. Type II Wald test for Sum of Squares was used to test the statistical significance of the main effects and interaction.

Post-hoc analysis for differences between the two groups at each stage employed Bonferroni’s multiple comparisons test. Post-hoc pairwise comparisons were performed on the log odds-ratio scale. Responses from the ordinal model (on the linear scale) were back transformed to estimates of the probability distribution of each rating and the average of these probability distributions (mean class) was plotted along with the standard errors (Figure 1).

In addition, a cumulative ordinal logistic regression analysis was carried out to assess the relationship of pain during manipulation as the dependent variable, with the independent variables of age, gender, and operator, baseline level of pain, fracture type and Frykman classification. . The regression model (Table 3) yielded an R^2^ of 46.4% for the overall regression. Individual p-values for each of the predictors along with the odds ratio and 95% Confidence Intervals (95% CI) are reported in Table 3. Diagnostics confirmed the lack of multicollinearity using the Variance Inflation Factor (VIF). A p-value of less than 0.05 was considered to be statistically significant.

**Statistical analysis**

Data analysis was performed using Minitab 19 (Minitab LLC, PA USA) and Graphpad Prism 9. Descriptive statistics shown in Table 1 for the 25 participants in each group employ the median, interquartile range, frequency and percentage of patients as appropriate. Differences between HB and CPB groups on demographics, fracture type/classification and outcome variables were tested using chi-squared tests (*χ* ^2^) for categorical variables and the Mann-Whitney test for age due to its non-parametric distribution (Table 1). Mixed ordinal logistic regression (using logit link) was used to test for differences between the two techniques, between the three stages (baseline, injection, manipulation), as well as the interaction of the two main effects (Stage x Method) (Table 2). Mixed ordinal regression was used to take into account the ordinal nature of the VAS score and the correlation of VAS values obtained from each patient. Stage and method were included as main effects and patients were included as a random effect. Type II Wald test for Sum of Squares was used to test the statistical significance of the main effects and interaction.

Post-hoc analysis for differences between the two groups at each stage employed Bonferroni’s multiple comparisons test. Post-hoc pairwise comparisons were performed on the log odds-ratio scale. Responses from the ordinal model (on the linear scale) were back transformed to estimates of the probability distribution of each rating and the average of these probability distributions (mean class) was plotted along with the standard errors (Figure 1).

In addition, a cumulative ordinal logistic regression analysis was carried out to assess the relationship of pain during manipulation as the dependent variable, with the independent variables of age, gender, and operator, baseline level of pain, fracture type and Frykman classification. . The regression model (Table 3) yielded an R^2^ of 46.4% for the overall regression. Individual p-values for each of the predictors along with the odds ratio and 95% Confidence Intervals (95% CI) are reported in Table 3. Diagnostics confirmed the lack of multicollinearity using the Variance Inflation Factor (VIF). A p-value of less than 0.05 was considered to be statistically significant.

**Results**

Between January 2021 and June 2021, a total of 50 patients met the inclusion criteria and were consecutively recruited and randomized to receive either a HB or CPB (Figure 1.). Twenty-five patients were included evenly in both groups. Six patients were excluded for not meeting the inclusion criteria; 4 were excluded for ipsilateral upper limb fractures, 2 excluded due to the presence of significant head trauma.

- Insert Figure 1 here -

*Figure 1 – CONSORT Flowchart displaying enrolment, allocation, follow up and analysis of participants.*

The descriptive characteristics of our study population are displayed in [**Table 1**]. Out of the 50 patients, the majority were males (66%) who sustained isolated distal end radius (DER) fractures (84%). The age range was 13-67 years old. Frykman classification 1 and 3 were the most common patterns encountered. The vast majority of the fractures underwent non-operative treatment after successful reduction (88%) with no significant difference between block method and outcome. Nine patients (18%) underwent operative management; 5 with percutaneous pinning and 4 with ORIF. There were no documented complications for either technique. Two patients; one in each group, subsequently required re-manipulation. No statistical significances were observed between the distributions and end-outcomes of both groups, thereby ensuring comparability.

Table 1 Distribution and between-group comparisons of participants according to patient and fracture variables

| **Variable** |  | **Hematoma Block n (%)** | **Circumferential Block n (%)** | **χ2; p-value/MWU; p-value** |
| --- | --- | --- | --- | --- |
| **Age** | Median (IQR) | 41 (30.5–51.5) | 44 (29.5–53) | MWU: 307, p = 0.9195 |
| **Sex** | Male | 15 (60%) | 18 (72%) | χ2: 0.3565, p = 0.5505 |
|  | Female | 10 (40%) | 7 (28%) |  |
| **Fracture** | BB | 3 (12%) | 5 (20%) | χ2: 0.1488, p = 0.6997 |
|  | DER | 22 (88%) | 20 (80%) |  |
| **Frykman** | 1 | 13 (52%) | 11 (44%) | χ2: 3.892, p = 0.4208 |
|  | 2 | 1 (4%) | 0 (0%) |  |
|  | 3 | 9(36%) | 8 (32%) |  |
|  | 4 | 1 (4%) | 5 (20%) |  |
|  | 8 | 1 (4%) | 1 (4%) |  |
| **Radiograph** | Accepted | 23 (92%) | 21 (84%) | χ2: 0.1894, p = 0.6634 |
|  | Reduced | 2 (8%) | 4 (16%) |  |
| **Definitive treatment** | Conservative | 22 (88%) | 19 (76%) | χ2: 0.5420, p = 0.4616 |
|  | Operative | 3 (12%) | 6 (24%) |  |
| **Need for Re-manipulation** | No | 24 (96%) | 24 (96%) | χ2: 0.000, p > 0.9999 |
|  | Yes | 1 (4%) | 1 (4%) |  |
| **Complications** | No | 25 (100%) | 25 (100%) | χ2: 0.000, p > 0.9999 |
|  | Yes | 0 (0%) | 0 (0%) |  |

*Table 2 Results of mixed ordinal logistic regression measuring pain scores across stages of anesthesia*

| **Effect** | **LR χ2 (df)** | **P value** |
| --- | --- | --- |
| Stage x Method | **χ2** (2) = 29.64 | <0.0001 |
| Stage | **χ2** (2) = 187.87 | <0.0001 |
| Method | **χ2** (1) = 5.79 | = 0.02 |

Mixed ordinal logistic regression (Table 2.) revealed significant main effects for both method and stage (P: 0.02, <0.0001 respectively), the latter being an indicator of achieved analgesia regardless of method. In addition, the significant interaction effect (P<0.0001) indicates that the differences between block methods vary across the stages of analgesia. Post-hoc comparisons using a Bonferroni correction confirmed that a difference between block methods is only found during the manipulation stage (P<0.0001).


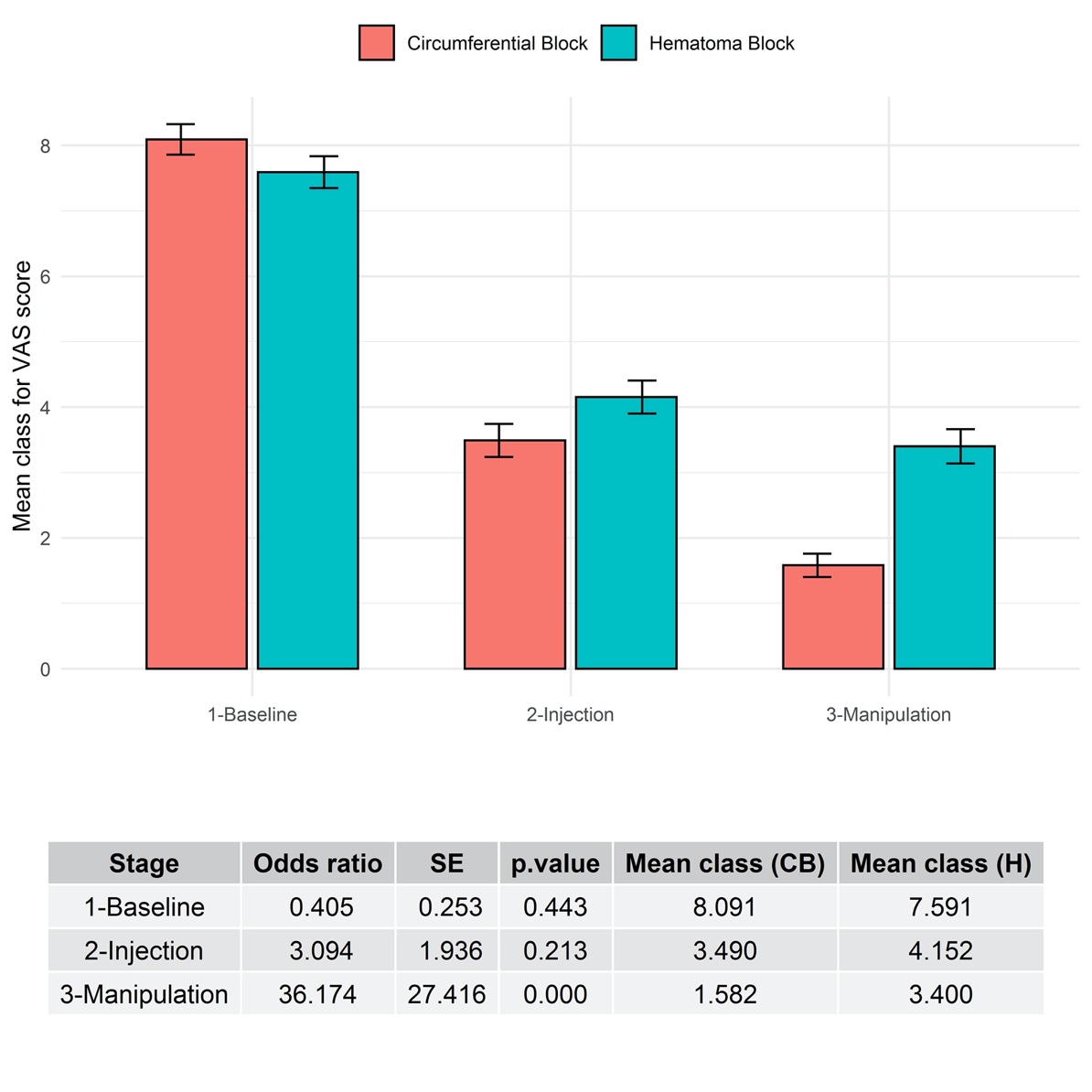


Figure 2 – Pain scores across the different stages of management; baseline, injection and manipulation

Post-hoc analysis revealed that the only significant difference between the two groups was found to be during the manipulation stage (p = <0.0001). The lack of a difference at baseline confirms the comparability of both groups. There was no difference during the injection stage (p = 0.213). The mean pain score during manipulation for HB was 2.44 and for CPB 0.64 (**Figure 2**). Hematoma blocks had a wider VAS range of 0-5 (SD = 1.44), whereas CPB had a narrower VAS range of 0-3 (SD = 0.86).

Table 3 – Ordinal logistic regression for predictors of pain during manipulation (BB; Both bone/ DER; Distal end radius)

| **Variable** | **OR** | **95% CI** | **P** |
| --- | --- | --- | --- |
| **Age** [years] | 1.04 | 1.00 – 1.09 | **0.050*** |
| **Gender** [F/M] | 0.97 | 0.30 – 3.14 | 0.953 |
| **Fracture** [BB/DER] | 2.66 | 0.39 – 18.10 | 0.318 |
| **Frykman Classification** [vs. 1] |  |  |  |
| 2 | 3.88 | 0.18 – 82.44 | 0.384 |
| 3 | 0.65 | 0.21 – 2.02 | 0.455 |
| 4 | 0.20 | 0.03 – 1.24 | 0.083 |
| 8 | 1.51 | 0.15 – 15.42 | 0.729 |
| **Baseline Pain Score** | 1.74 | 0.99 – 3.05 | **0.05*** |
| **Operator** [A/B] | 1.63 | 0.51 – 5.27 | 0.411 |
| **Block Method** [HB/ CPB] | 21.24 | 5.60 – 80.54 | **<0.001*** |
| OR: Odds ratio; SE: Standard error; CI: Confidence interval | | | |

Predictors of pain during manipulation using multivariate regression analysis are displayed in Table 3. None of gender, fracture types and Frykman classification were found to be significant factors in affecting severity of pain during reduction. Notably, there was no significant operator variability in pain among block methods. Significant predictors of pain during manipulation include; increasing age (p =0.05), baseline pain score (p = 0.05), and block method (p <0.0001). Overall, the odds of having a higher pain grade at the manipulation stage were 21.24 times higher in the HB group than the CPB group (OR = 21.24, P < 0.001), whilst keeping all other variables constant.
